# The causal relationships between inflammatory cytokines, blood metabolites, and thyroid cancer: a two-step Mendelian randomization analysis

**DOI:** 10.1007/s12672-025-02029-w

**Published:** 2025-03-12

**Authors:** Weihao Liu, Yuxiao Sun, Yifei Zhang, Detao Yin

**Affiliations:** 1 Department of Thyroid Surgery, The First Affiliated Hospital of Zhengzhou University, Zhengzhou, 450052 China; 2 Engineering Research Center of Multidisciplinary Diagnosis and Treatment of Thyroid Cancer of Henan Province, Zhengzhou, 450052 China

**Keywords:** Inflammatory cytokine, Blood metabolites, Thyroid cancer, Mendelian randomization, Causal effect, Genetics

## Abstract

**Background:**

Thyroid cancer is a prevalent malignant tumor, especially with a higher incidence in women. Tumor microenvironment changes induced by inflammation and alterations in metabolic characteristics are critical in the development of thyroid cancer. Nevertheless, their causal relationships remain unclear.

**Methods:**

We utilized thyroid cancer GWAS data from the Global Biobank Meta-Analysis Initiative and GWAS data of 91 inflammatory cytokines and 1400 blood metabolites obtained from the GWAS Catalog to evaluate the causality between inflammatory cytokines, blood metabolites, and thyroid cancer using Mendelian randomization (MR). Initially, we identified inflammatory cytokines having a significant causal effect on thyroid cancer. Subsequently, for the identified positive blood metabolites, we applied a two-step mediation MR method to examine their mediating role in the causal effect of specific inflammatory cytokines on thyroid cancer.

**Results:**

Our forward MR analysis identified suggestive associations between 7 inflammatory cytokines and thyroid cancer risks, and found that tumor necrosis factor ligand superfamily member 14 (TNFSF14) (IVW-OR: 1.25, 95% CI 1.10–1.42, p = 0.0004) is a significant risk factor in thyroid cancer, and this causal relationship remained significant after Bonferroni correction. The reverse MR analysis identified suggestive causal associations between thyroid cancer and 3 inflammatory cytokines and ruled out the reverse causality between TNFSF14 and thyroid cancer. Then, we identified suggestive associations between 35 blood metabolites and 24 blood metabolite ratios with thyroid cancer, and found that 5-hydroxymethyl-2-furoylcarnitine (IVW-OR: 1.38, 95% CI 1.19–1.61, p = 0.00003) is a significant risk factor for thyroid cancer, with this causality remaining significant after Bonferroni correction. Finally, our two-step MR analysis indicated that Lactosyl-N-palmitoyl-sphingosine (d18:1/16:0) and X-12013 have a mediating effect in the causal relationship between TNFSF14 and thyroid cancer, with mediation proportions of 8.55% and 5.78%, respectively. Our MR analysis did not identify significant heterogeneity or horizontal pleiotropy.

**Conclusion:**

This study identified some inflammatory cytokines and blood metabolites associated with thyroid cancer risk and revealed the mediating role of specific blood metabolites between TNFSF14 and thyroid cancer, highlighting the critical role of inflammatory and metabolic pathways in the pathogenesis of thyroid cancer.

**Supplementary Information:**

The online version contains supplementary material available at 10.1007/s12672-025-02029-w.

## Introduction

Thyroid cancer significantly contributes to the global cancer burden, as highlighted by the World Health Organization. In 2020, there were 586,000 new cases reported globally, ranking it 9th overall and 5th among female patients [1]. Previous studies have shown that thyroid cancer involves complex mechanisms, with substantial evidence linking it to immune system dysfunction. Recent evidence has confirmed that inflammation participates in the etiology of thyroid cancer, with the tumor microenvironment being crucial [2, 3]. At the same time, with the advancement of metabolomics, the technology for studying thyroid cancer samples by identifying and analyzing specific types of metabolites has gradually matured [4]. As the largest endocrine organ, the rising prevalence of cancers in thyroid significantly increases healthcare costs and the burden on social services. Therefore, it is urgent to gain insights into the complex mechanisms underlying thyroid cancer development to identify risk factors for its onset and to provide guidance for developing personalized therapies targeting specific immune mechanisms.

Inflammatory cytokines are produced by multiple cells and their biological activities are essential for regulating cellular immune responses [5]. Inflammatory cytokines are critical in the development and invasion of most cancer types [6–8]. Recently, the investigation of inflammatory cytokines and their receptors as treatment targets for cancer has garnered significant attention [9]. The identification of abnormal and dysregulated expression of inflammatory cytokines in various human cancers underscores the rationale behind this research [10]. As the largest human endocrine organ, the thyroid remains a persistent focus of research regarding the mechanisms and risk factors of thyroid cancer. Studies on inflammatory cytokines and their connection to thyroid cancer are continuously emerging. One study has demonstrated that interleukin-6 activates the MAPK and JAK pathways in papillary thyroid cancer cells, leading to their dedifferentiation [11]. Additionally, METTL3 inhibits the progression of papillary thyroid cancer by recruiting neutrophils through the m6A/c-Rel/IL-8 pathway [12]. In summary, substantial evidence indicates that certain inflammatory cytokines are linked to the risks of thyroid cancer, but the findings are scattered and inconsistent. For instance, while research has shown that interleukin-6 can induce dedifferentiation in papillary thyroid carcinoma, other studies have found that interleukin-6 expression levels decrease in specific anaplastic thyroid cancer cell lines [13]. In summary, current research on the association between inflammatory cytokines and thyroid cancer is insufficient.

Recent studies also indicate that the metabolic characteristics and preferences of tumors change during cancer progression [14], and metabolic changes can regulate inflammatory responses and tumor microenvironments [15]. The Warburg effect, characterized by preferences for glycolysis and lactate secretion even with oxygen, exemplifies a metabolic property regulated by oncogenes in various proliferating tumors [16]. Existing studies have indicated that citric acid and lactic acid are the most important tumor metabolites in thyroid cancer [17]. Meanwhile, lactic acid has been proven to increase cancer invasiveness in some types of cancer by promoting the polarization of tumor-associated macrophages [18]. It can be speculated that the interaction between specific inflammatory cytokines and metabolites is of great significance for shaping the tumor microenvironment, but currently, related research is quite scarce.

In Mendelian randomization (MR), genetic variations as instrumental variables (IVs), are utilized to mimic randomization and reduce confounding in observational studies [19, 20]. This method is less susceptible to confounders as genetic variation is randomly allocated during meiosis. This random allocation allows for precise capture of exposure without being impacted by environmental factors or reverse causation [21, 22]. Consequently, we employed MR analysis to investigate the potential causal relationships between circulating inflammatory cytokines, blood metabolites, and thyroid cancer risk. Initially, specific inflammatory cytokines demonstrating causal relationships with thyroid cancer were identified. Subsequently, a two-step mediation MR analysis was conducted to investigate the potential mediating role of blood metabolites in the causal pathway between inflammatory cytokines and thyroid cancer, and to quantify the proportion of the mediation effect, thereby establishing a potential pathogenic pathway from circulating inflammatory cytokines through blood metabolites to thyroid cancer.

## Materials and methods

### Study design

**Fig. 1:**
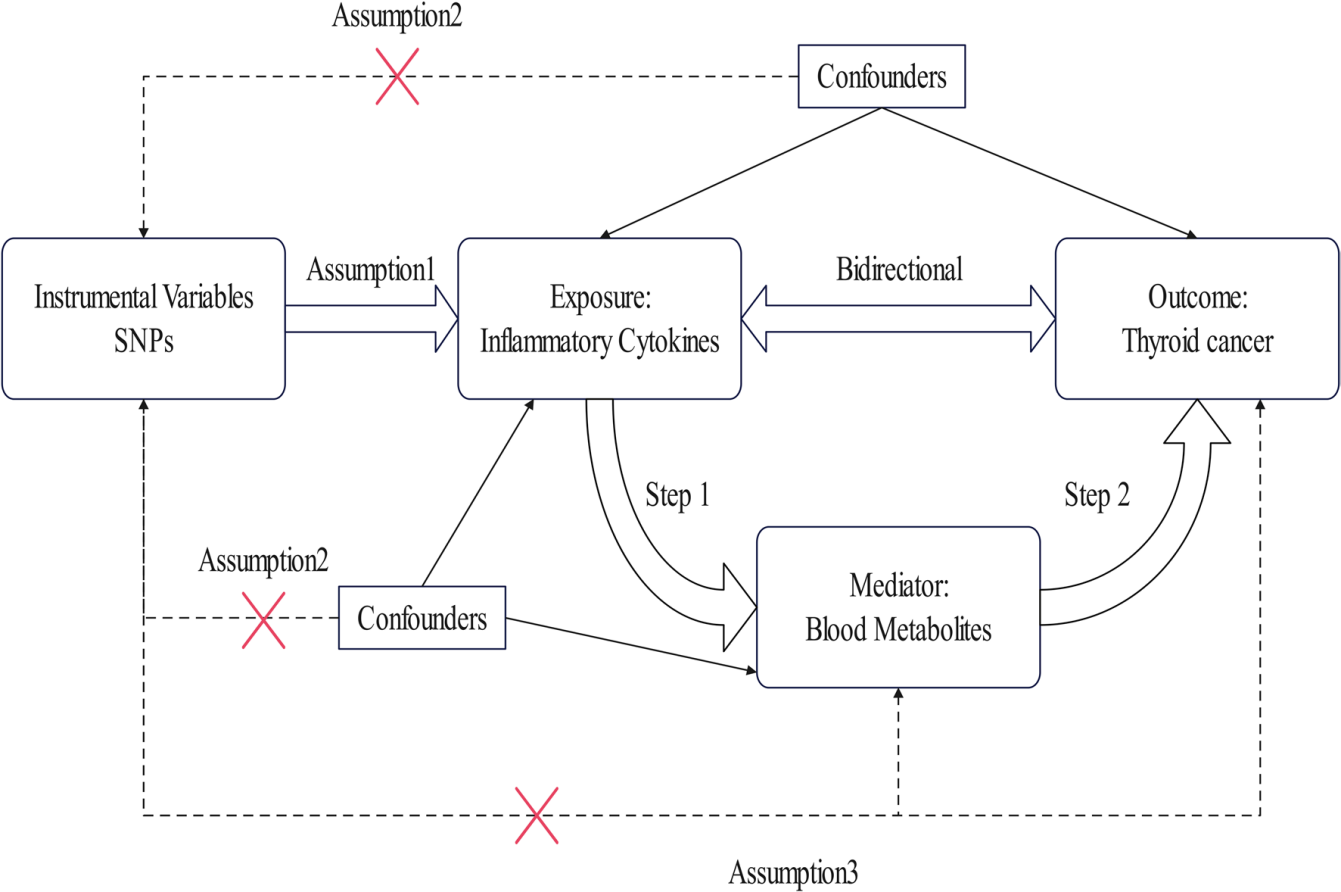
Study design overview. (SNP: single-nucleotide polymorphism)

We examined the causality between 91 inflammatory cytokines, 1,400 blood metabolites and thyroid cancer. MR analysis is a technique to reveal causal links between exposure and outcome based on genetic variations of exposures. The IVs must satisfy the assumptions: (1) the genetic variation must be strongly correlated with the exposure factor, (2) the genetic variation is independent of confounding factors, (3) the genetic variation must impact the outcome solely via the exposure pathway. Figure 1 presents the flowchart of our MR processes.

### Data sources

Data from the Global Biobank Meta-Analysis Initiative (GBMI) were utilized to perform genome-wide association study (GWAS) meta-analyses on thyroid cancer among Europeans, including 6,015 cases and 1,333,754 controls. The GBMI project highlights the benefits gained from the collaborative efforts of 23 global biobanks, which advance genetic discoveries by providing larger sample sizes and increased ancestral diversity [23]. Thyroid cancer was defined using the International Classification of Diseases, specifically Phecode 193, with the codes V10.87, C73, and Z85.850. Data on inflammatory cytokine levels were from a GWAS conducted across 11 groups, involving 14,824 Europeans [24]. After excluding any missing data, the Olink Targeted Inflammation Panel was employed to assess plasma cytokine levels, covering 91 inflammatory cytokines. Finally, the data on 1,400 blood metabolites were from a GWAS involving 8,299 European participants, encompassing 1091 blood metabolites and 309 metabolite ratios [25].

### Choosing instrumental variables

We adopted a statistically lenient threshold of p < 5 × 10^–6^ to identify single nucleotide polymorphisms (SNPs) associated with 91 circulating inflammatory cytokines and 1,400 blood metabolites to capture a broader range of genetic variation [26, 27]. These SNPs served as potential IVs for the study. Linkage disequilibrium (LD) is the non-random occurrence of genetic variants in the population, resulting from inherited chromosomal segments of genetic material, such that alleles for genetic variants close together are always co-inherited unless randomly segregated by recombination. To minimize LD between IVs in our MR analysis, we implemented a clumping procedure using the European 1000 Genomes reference panel within a 10,000-kb window, eliminating SNPs that exhibited pairwise LD with r^2^ > 0.001. Palindromic SNPs with a minor allele frequency of 0.42 or greater were excluded due to their ambiguous nature. To ensure the IVs were strongly associated with circulating inflammatory cytokines, we retained IVs with an F-statistic > 10 [28]. The F-statistic is equal to R^2^ × (N-2)/(1-R^2^), where R^2^ represents the proportion of variance in the exposure variable, and N represents the sample size of exposures. The R^2^ is equal to 2 × (1-MAF) × MAF × β^2^, where MAF is the minor allele frequency, and β is the effect size of the SNP on exposures. In the subsequent reverse MR analysis, our selection criteria remained unchanged, but the threshold was tightened from p < 5 × 10^–6^ to p < 5 × 10^–8^ to achieve genome-wide significance.

### Statistical analysis

The R 4.3.1 was utilized for MR analysis and data visualization, we utilized R packages such as “TwoSample MR” and “MR-PRESSO.” To examine the causality between inflammatory cytokines and risks of thyroid cancer, a bidirectional MR analysis was first employed. To accurately examine causal effects, we used multiple MR detection methods, including the Inverse-Variance Weighted (IVW), MR-Egger, weighted median, simple mode, and weighted mode. When the assumptions are met, namely that all instrumental variables are valid and there is no horizontal pleiotropy, the IVW method can synthesize information from all instrumental variables to estimate the causal effect through weighted averaging. Therefore, we use the IVW method as the main approach for MR analysis [29, 30]. Cochran's Q test was employed to test heterogeneity [31]. The primary factor influencing the validity of Mendelian randomization analysis results is pleiotropy, particularly horizontal pleiotropy, which arises when genetic variants influence outcomes via pathways unrelated to the risk factor of interest, violating the third assumption of Mendelian randomization analysis. The MR-PRESSO method assesses overall horizontal pleiotropy across all instrumental variables in a single Mendelian randomization test by contrasting the observed residual sum of squares of all variants with the expected distance under the null hypothesis of no horizontal pleiotropy [32, 33]. The MR-Egger intercept represents the average pleiotropic effect across the genetic variants (the average direct effect of a variant on the outcome). If the intercept is significantly different from zero, it indicates the presence of directional pleiotropy [34]. Therefore, we evaluated horizontal pleiotropy through the MR-Egger intercept and MR-PRESSO test. The leave-one-out method was utilized to evaluate the impact of individual genetic loci on total estimates [35]. Scatter and funnel plots were generated to visualize and analyze the results.

To eliminate false positive results and significantly enhance the credibility of the study conclusions, we implemented a strict Bonferroni correction [36]. Following the Bonferroni correction, the significance threshold was set at 5.49 × 10^–4^ and 3.57 × 10^–5^. In cases where the p-value is less than 0.05 but higher than the Bonferroni-corrected threshold, it is considered evidence of a potential association [37–39].

Finally, in order to explore the roles of blood metabolites and inflammatory cytokines on thyroid cancer, we conducted a mediation MR analysis, which helps us evaluate the mediating role in the causal pathways between exposure factors and outcomes. We used the classic two-step mediation MR analysis. Firstly, we analyzed the causal effect value β0 of 91 inflammatory cytokines on thyroid cancer and performed a reverse MR analysis to rule out the reverse causal effects of significant inflammatory cytokines identified in the forward analysis. In the second step, we sequentially evaluated the causality between 1400 blood metabolites and thyroid cancer, as well as between specific inflammatory cytokines and specific blood metabolites. We then calculated the causal effect β1 between specific inflammatory cytokines and blood metabolites, and the causal effect β2 between specific blood metabolites and thyroid cancer, using the product of β1 and β2 as the mediation effect value β3. We divided β3 by β0 to derive the mediation effect proportion [40–42]. In these two-step analyses, we still employed multiple MR methods, with the IVW method as the primary approach. We used Cochran's Q test to assess heterogeneity and the MR-Egger intercept test to evaluate horizontal pleiotropy. By using two-sample and mediation MR methods, we obtained more thorough understandings of the causalities and potential mechanisms between blood metabolites, inflammatory cytokines, and thyroid cancer.

## Results

### Selection of IVs

Information on the selected SNPs of inflammatory cytokines, blood metabolites and thyroid cancer are in the Supplementary Table 1–3.

### Causal effects of inflammatory cytokines on thyroid cancer

**Fig. 2:**
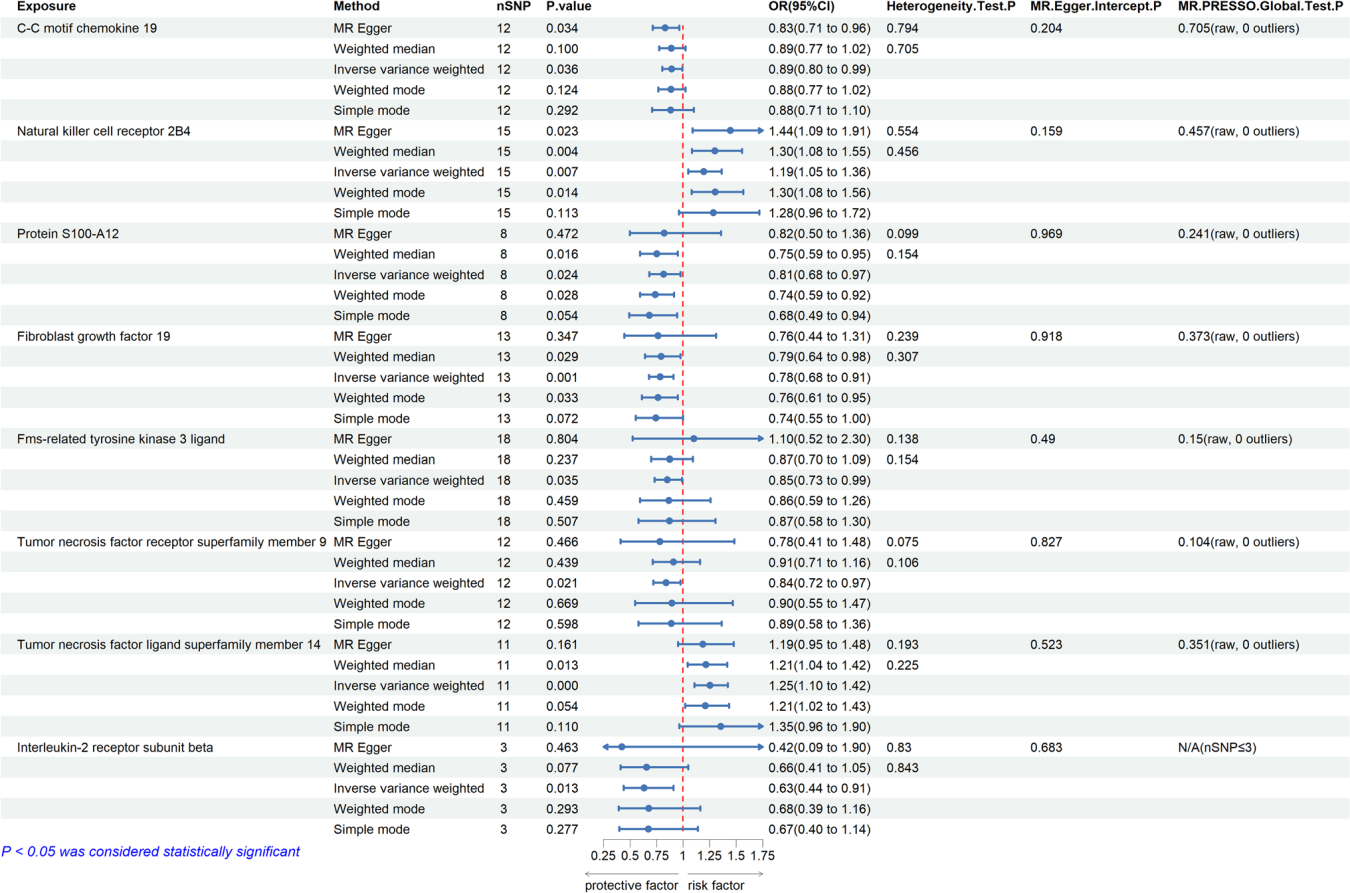
Positive Findings of causal effects of inflammatory cytokines on thyroid cancer. (nSNP: number of single-nucleotide polymorphism. OR: odd ratio.CI: Confidence Interval)

We have comprehensively recorded results of the forward MR analysis in the Supplementary Table 4. To enhance the readability of the article, we have plotted the positive analysis results in Fig. 2. There is a suggestive association between natural killer cell receptor 2B4 (CD244) (IVW-OR: 1.19, 95% CI 1.05–1.36, p = 0.007) and an increased risk of thyroid cancer. Genetically predicted fibroblast growth factor 19 (FGF19) (IVW-OR: 0.78, 95% CI 0.68–0.91, p = 0.001), interleukin-2 receptor subunit beta (IL2RB) (IVW-OR: 0.63, 95% CI 0.44–0.91, p = 0.013), tumor necrosis factor receptor superfamily member 9 (TNFRSF9) (IVW-OR: 0.83, 95% CI 0.72–0.97, p = 0.021), protein S100-A12 (S100-A12) (IVW-OR: 0.81, 95% CI 0.68–0.97, p = 0.024), Fms-related tyrosine kinase 3 ligand (FLT3LG) (IVW-OR: 0.85, 95% CI 0.73–0.99, p = 0.035), and C–C motif chemokine 19 (CCL19) (IVW-OR: 0.89, 95% CI 0.80–0.99, p = 0.036) are suggestively associated with decreased risks of thyroid cancer. The IVW method revealed that genetically determined higher levels of tumor necrosis factor ligand superfamily member 14 (TNFSF14) (one-SD increase) were significantly associated with 25% higher odds of thyroid cancer (IVW-OR: 1.25, 95% CI 1.10–1.42, p = 0.0004). This finding was consistent with the weighted median method (OR: 1.21, 95% CI 1.04–1.42, p = 0.013). It is important to note that our primary method is the IVW method, but we also displayed the results of several other analysis methods in Fig. 2.

**Fig. 3:**
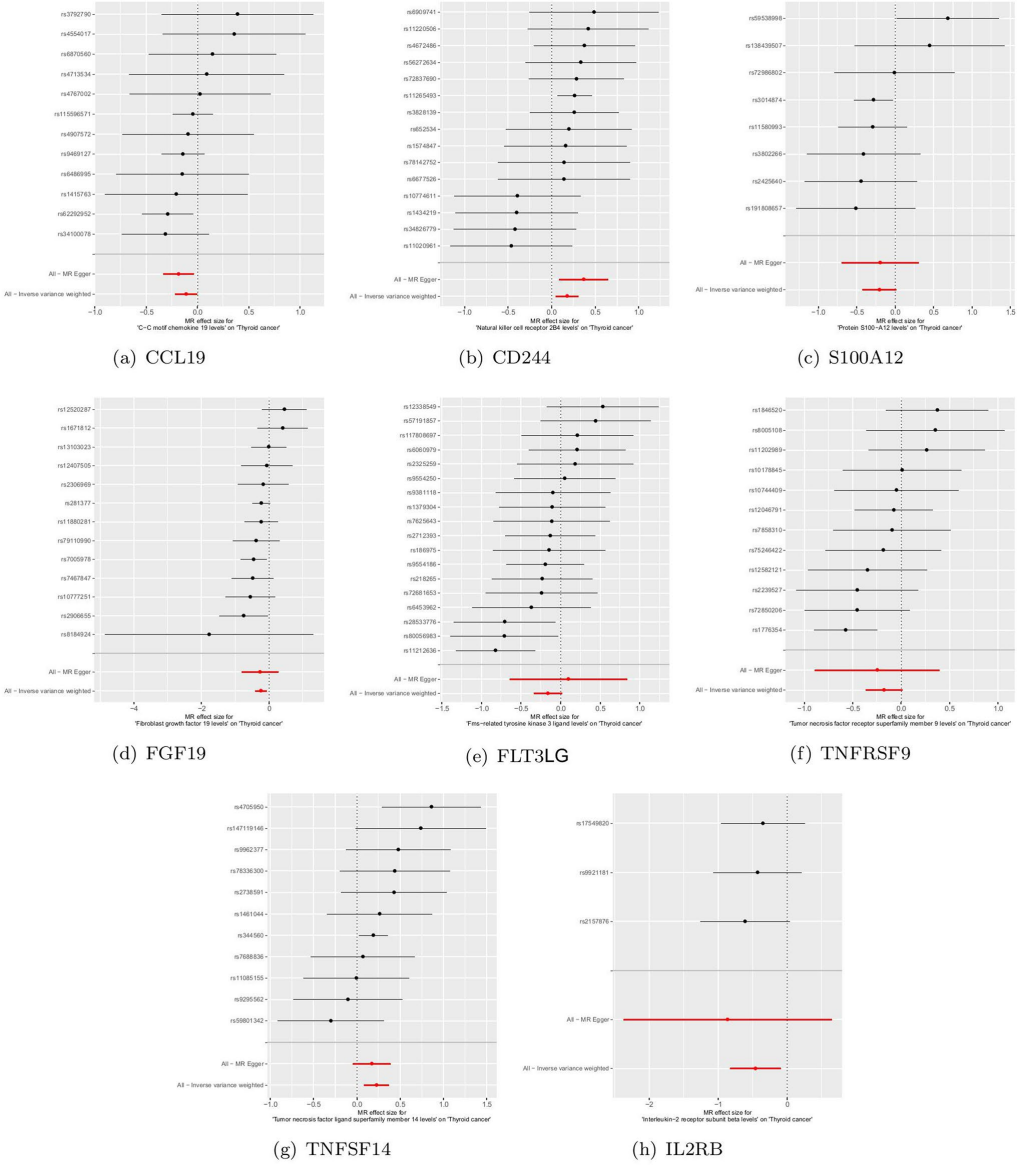
Forest plots of positive findings in Mendelian randomization analysis for inflammatory cytokines on thyroid cancer. **a** CCL19 on thyroid cancer. **b** CD244 on thyroid cancer. **c** S100A12 on thyroid cancer. **d** FGF19 on thyroid cancer. **e** FLT3LG on thyroid cancer. **f** TNFRSF9 on thyroid cancer. **g** TNFSF14 on thyroid cancer. **h** IL2RB on thyroid cancer (MR: Mendelian randomization. CCL19: C–C motif chemokine 19. CD244: Natural killer cell receptor 2B4. S100A12: Protein S100-A12. FGF19: Fibroblast growth factor 19. FLT3LG: Fms-related tyrosine kinase 3 ligand. TNFRSF9: Tumor necrosis factor receptor superfamily member 9. TNFSF14: Tumor necrosis factor ligand superfamily member 14. IL2RB: Interleukin-2 receptor subunit beta.)

**Fig. 4:**
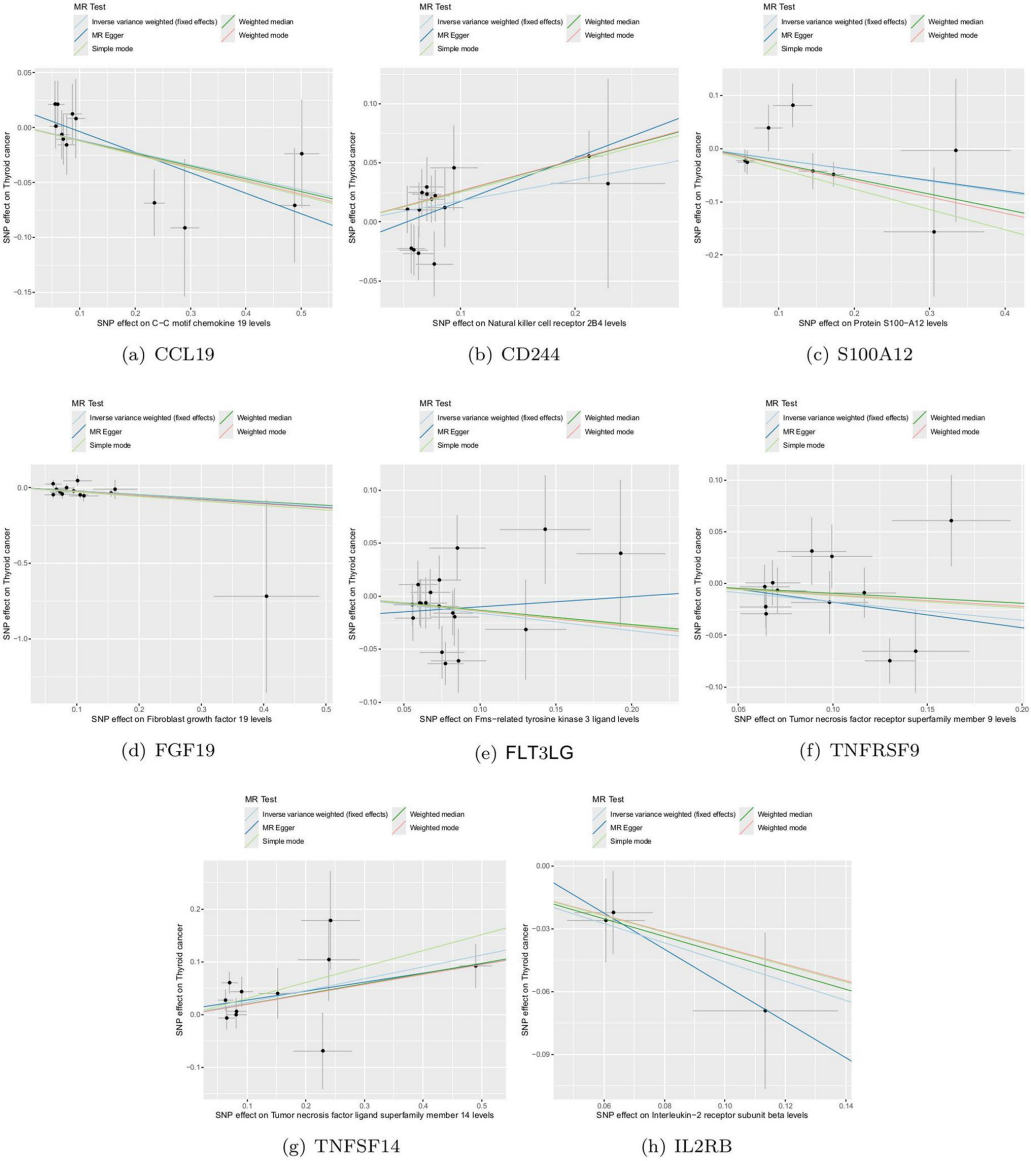
Scatter plots of positive findings in Mendelian randomization analysis for inflammatory cytokines on thyroid cancer. **a** CCL19 on thyroid cancer. **b** CD244 on thyroid cancer. **c** S100A12 on thyroid cancer. **d** FGF19 on thyroid cancer. **e** FLT3LG on thyroid cancer. **f** TNFRSF9 on thyroid cancer. **g** TNFSF14 on thyroid cancer. **h** IL2RB on thyroid cancer

**Fig. 5:**
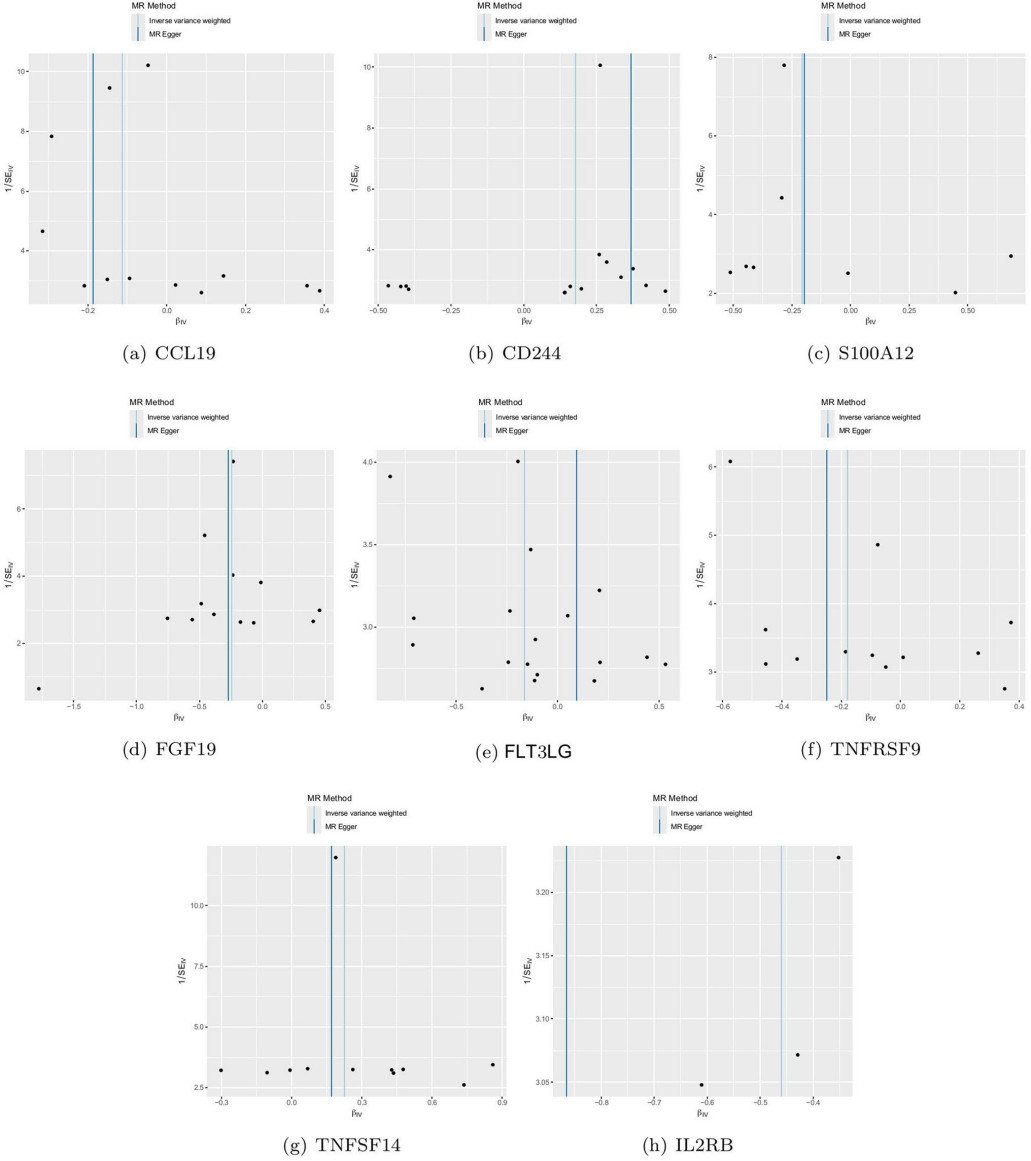
Funnel plots of positive findings in Mendelian randomization analysis for inflammatory cytokines on thyroid cancer. **a** CCL19 on thyroid cancer. **b** CD244 on thyroid cancer. **c** S100A12 on thyroid cancer. **d** FGF19 on thyroid cancer. **e** FLT3LG on thyroid cancer. **f** TNFRSF9 on thyroid cancer. **g** TNFSF14 on thyroid cancer. **h** IL2RB on thyroid cancer

**Fig. 6:**
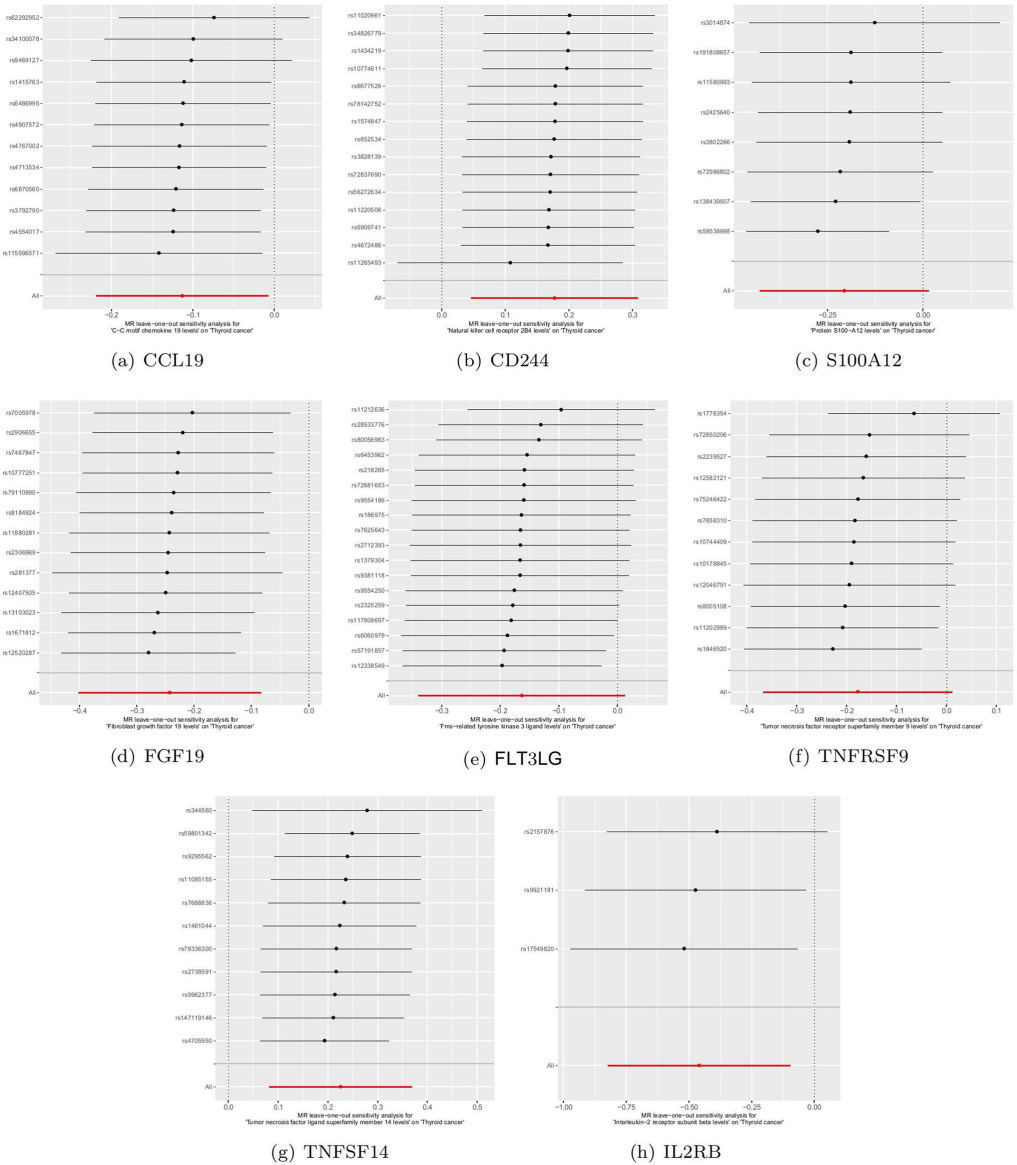
The Leave-one-out analyses of positive findings in Mendelian Randomization analysis for inflammatory cytokines and thyroid cancer. **a** CCL19 on thyroid cancer. **b** CD244 on thyroid cancer. **c** S100A12 on thyroid cancer. **d** FGF19 on thyroid cancer. **e** FLT3LG on thyroid cancer. **f** TNFRSF9 on thyroid cancer. **g** TNFSF14 on thyroid cancer. **h** IL2RB on thyroid cancer

For the positive analysis results mentioned above, our heterogeneity tests did not detect significant heterogeneity between the outcomes and exposures analyzed (all Cochran’s Q p > 0.05) and relevant results are comprehensively recorded in Supplementary Table 9–10. The MR-PRESSO method also identified no outlier SNPs (all p > 0.05). Additionally, the MR-Egger intercept indicated no horizontal pleiotropy. Figures 3a-h, 4a-h, 5a-h and 6a-h display forest, scatter, as well as funnel plots, and leave-one-out analyses for sensitivity analyses. We should point out that when analyzing the causal effect of interleukin-2 receptor subunit beta on thyroid cancer, the number of valid SNPs extracted was too small. Therefore, we did not perform MR-PRESSO analysis on this positive result. However, the MR-Egger p-value (> 0.05) suggests no horizontal pleiotropy in this subgroup analysis.

### Causal effects of thyroid cancer on inflammatory cytokines

**Fig. 7:**
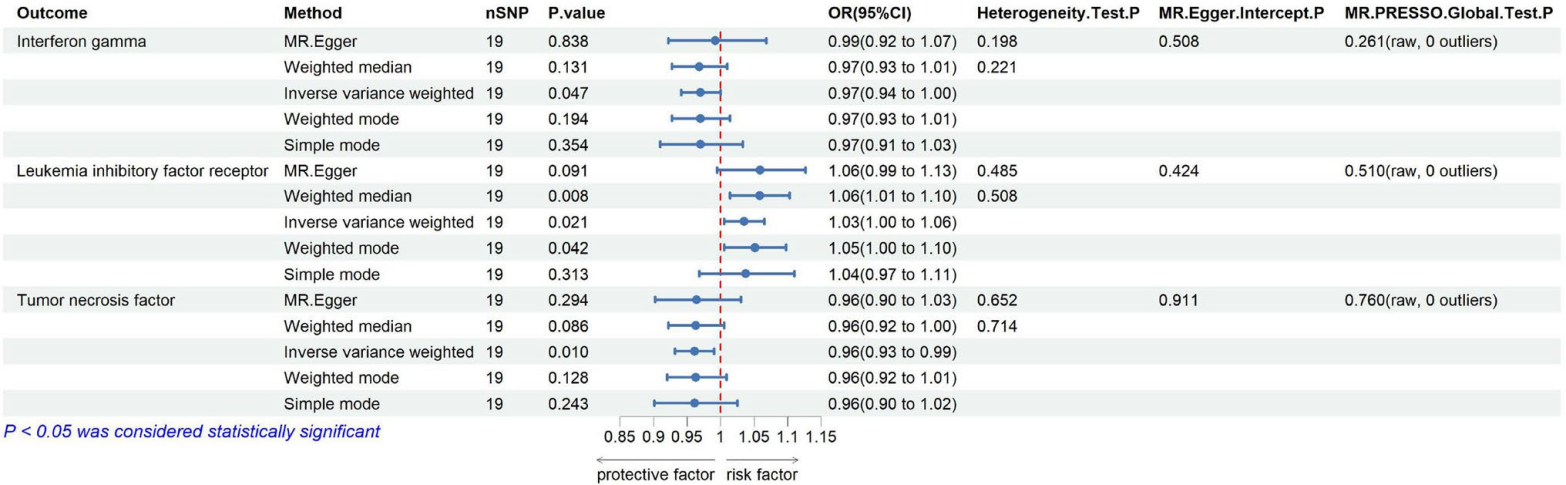
Positive Findings of causal effects of thyroid cancer on inflammatory cytokines (nSNP: number of single-nucleotide polymorphism. OR: odd ratio.CI: Confidence Interval)

During the reverse MR analysis, we were able to easily extract sufficient numbers of valid SNPs, even we strictly tightened the p-value for SNP selection to < 5 × 10^–8^. We have comprehensively recorded the reverse MR analysis results in the Supplementary Table 5 and plotted the positive results in Fig. 7. Our research results indicate that thyroid cancer is correlated with decreased circulating TNF levels (IVW-OR: 0.96, 95% CI 0.93–0.99, p = 0.009) and IFN-γ (IVW-OR: 0.97, 95% CI 0.94–1.00, p = 0.046), as well as a suggestive association with increased circulating leukemia inhibitory factor receptor (LIFR) levels (IVW-OR: 1.03, 95% CI 1.01–1.06, p = 0.020). For the reverse MR analysis results, our sensitivity analyses revealed no significant heterogeneity or horizontal pleiotropy and the relevant results are comprehensively recorded in Supplementary Table 11–12. The MR-PRESSO method also did not identify any outlier. It is important to emphasize that we found no reverse causal relationships of thyroid cancer on TNFSF14, which excludes the possibility that thyroid cancer directly causes changes in TNFSF14 levels. This strongly supports the unidirectional causal role of TNFSF14 on thyroid cancer and facilitates subsequent mediation MR analysis.

### Causal effects of blood metabolites on thyroid cancer

**Fig. 8:**
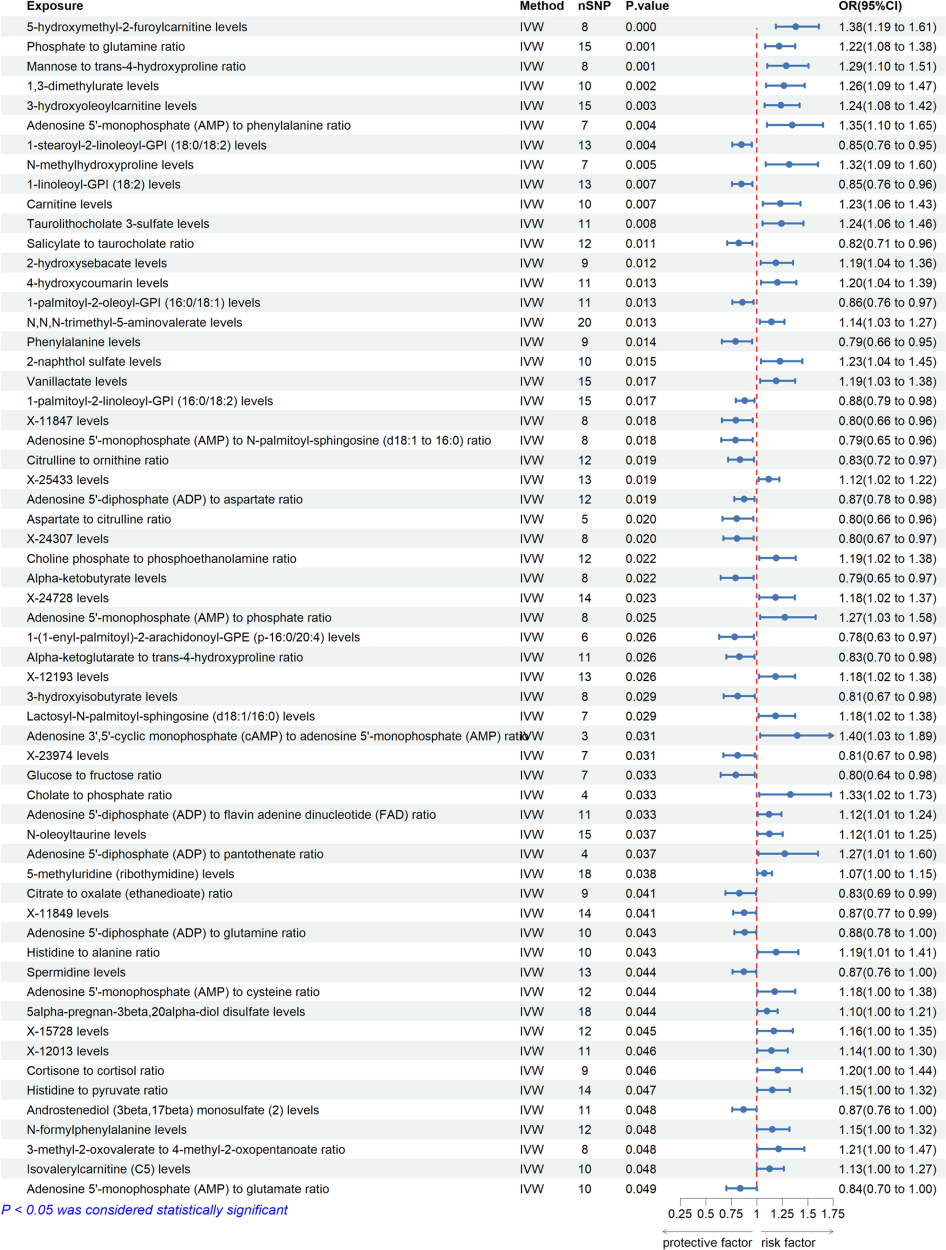
Positive Findings of causal effects of blood metabolites on thyroid cancer (nSNP: number of single-nucleotide polymorphism. OR: odd ratio.CI: Confidence Interval)

We applied the MR method to assess the causalities between 1400 blood metabolites and thyroid cancer, identifying 36 blood metabolites and 24 blood metabolite ratios associated with thyroid cancer risk. We illustrated these positive results in Fig. 8 and documented the complete analysis results in Supplementary Table 7. It is noteworthy that the IVW method revealed that genetically predicted higher levels of 5-hydroxymethyl-2-furoylcarnitine (one standard deviation increase) are associated with a 38% higher risk of thyroid cancer (IVW-OR: 1.38, 95% CI 1.19–1.61, p = 3 × 10^–5^). Concurrently, we performed the MR-Egger intercept test and Cochran's Q test, and the relevant results were documented in Supplementary Table 13–14.

### Causal effects of TNFSF14 on blood metabolites

**Fig. 9:**
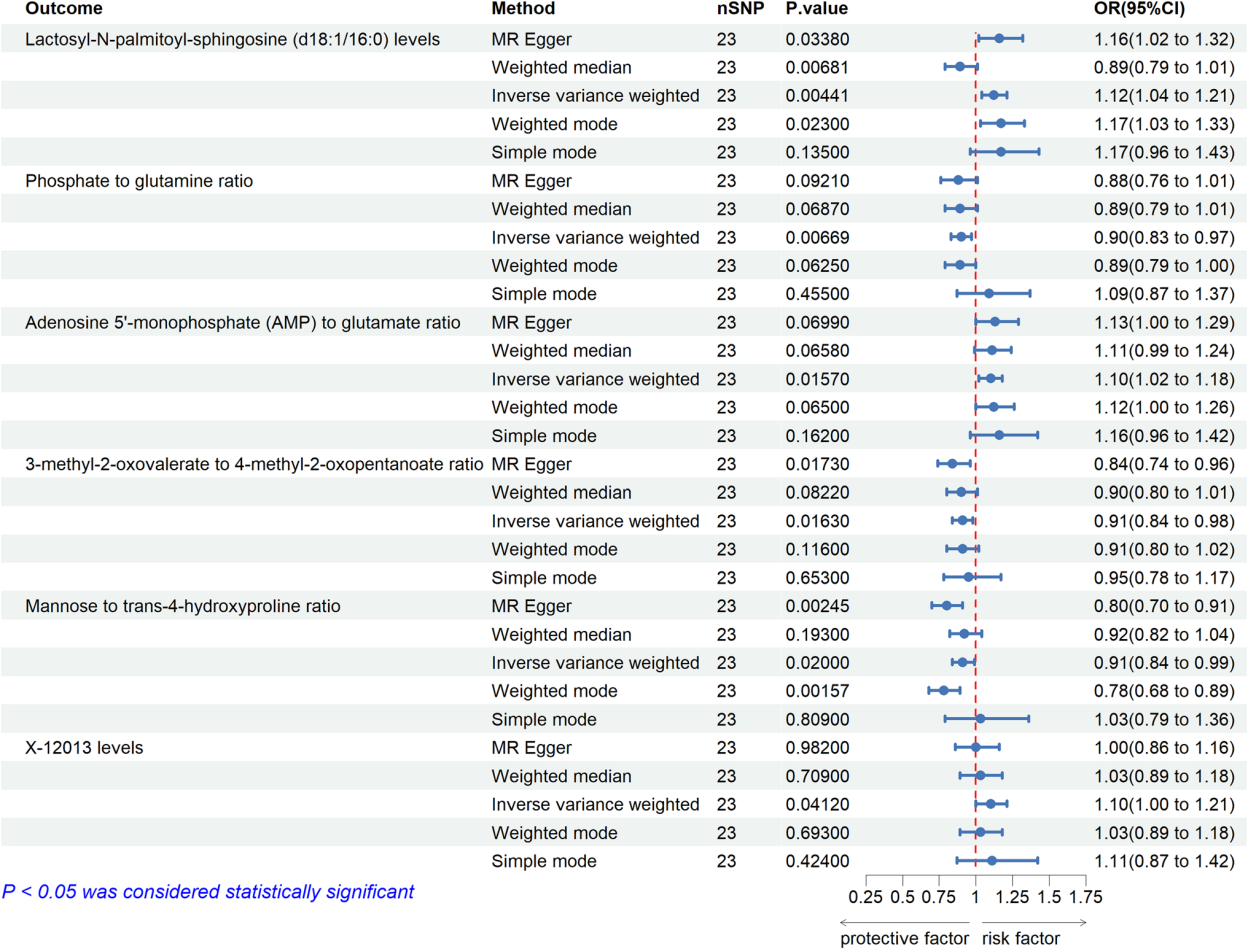
Positive Findings of causal effects of TNFSF14 on specific blood metabolites (nSNP: number of single-nucleotide polymorphism. OR: odd ratio.CI: Confidence Interval)

In previous analyses, we identified a significant causality between TNFSF14 and thyroid cancer and excluded reverse causality, while also finding that 36 blood metabolites and 24 blood metabolite ratios are associated with thyroid cancer risk. Therefore, we decided to further investigate the mediating role and mediating effects of these blood metabolites between TNFSF14 and thyroid cancer. We continued to use the TNFSF14 as the exposure and 36 blood metabolites and 24 blood metabolite ratios as the outcomes, to evaluate their causal relationships and thereby establish the inflammatory cytokine-blood metabolite mediation pathway. At this step, the IVW method suggested causal associations between TNFSF14 and the levels of 2 blood metabolites and the ratios of 4 metabolites. We plotted these positive results in Fig. 9. Although the p-values of these findings were not below the Bonferroni-corrected threshold, three MR analysis methods indicated a positive correlation between TNFSF14 and Lactosyl-N-palmitoyl-sphingosine (d18:1/16:0) levels (IVW-OR: 1.12, p = 0.001; MR-Egger-OR: 1.16, p = 0.0338; Weighted median-OR: 1.17, p = 0.023) and a negative correlation with Mannose to trans-4-hydroxyproline ratio (IVW-OR: 0.91, p = 0.02; MR-Egger-OR: 0.8, p = 0.00245; Weighted mode-OR: 0.78, p = 0.00157). Finally, we recorded the complete MR analysis results and sensitivity analysis results in Supplementary Table 8, 15–16.

### Mediation effect of blood metabolites between TNFSF14 and thyroid cancer

To gain a deeper understanding of the causal effect of TNFSF14 on thyroid cancer, we conducted a mediation MR analysis. Ultimately, we identified two blood metabolites that mediated the causal effect of TNFSF14 on thyroid cancer. The TNFSF14 can increase the risk of thyroid cancer by raising Lactosyl-N-palmitoyl-sphingosine (d18:1/16:0) levels and X-12013 levels. We calculated the mediating effects and mediation proportions using the product method and illustrated the relevant results in Table 1.

**Table 1:** The mediation effect and proportion of blood metabolites by IVW method

Exposure	β1	Mediator	β2	Outcome	β1*β2	β0	Mediated Proportion
TNFSF14	0.115	Lactosyl-N-palmitoyl-sphingosine (d18:1/16:0) levels	0.168	Thyroid cancer	0.019	0.225	8.55%
TNFSF14	0.097	X-12013 levels	0.134	Thyroid cancer	0.013	0.225	5.78%

## Discussion

First, we employed bidirectional MR analysis to explore the causality between 91 inflammatory cytokines and thyroid cancer. The forward MR analysis results suggested potential associations between circulating CD244 levels and increased risks in thyroid cancer. Additionally, there was a notable association between levels of FGF19, IL2RB, TNFRSF9, S100-A12, FLT3LG, and CCL19 and a decreased risk of thyroid cancer. By utilizing stringent Bonferroni correction, we identified a significant association between TNFSF14 and an high risks of thyroid cancer. We then conducted a reverse MR analysis using SNPs of thyroid cancer selected with a stricter threshold. Our results indicated a suggestive association between thyroid cancer and decreased circulating levels of TNF and IFN-γ, as well as a suggestive association with increased circulating levels of LIFR. Overall, while investigating the causality between 91 inflammatory cytokines and thyroid cancer, we established a significant unidirectional causality between TNFSF14 and thyroid cancer. In our bidirectional MR analysis, sensitivity analyses revealed no significant horizontal pleiotropy or heterogeneity, and the MR-PRESSO method detected no outliers. Subsequently, we incorporated GWAS data of 1400 blood metabolites and explored whether these metabolites could potentially mediate the causal effect of TNFSF14 on thyroid cancer. Ultimately, we identified a significant association between 5-hydroxymethyl-2-furoylcarnitine and increased risks of thyroid cancer, and found that TNFSF14 can increase the risk of thyroid cancer by elevating Lactosyl-N-palmitoyl-sphingosine (d18:1/16:0) levels and X-12013 levels.

TNFSF of cytokine-like molecules includes 19 ligands, while the TNFRSF, which comprises proteins binding to these ligands, includes 29 related receptors. The interactions between them mediate the survival, proliferation, and differentiation of various cells [43]. TNFRSF9 is broadly expressed in human CD4 + and CD8 + T cells and serves as a crucial activating immune checkpoint molecule in T cells. On one hand, TNFRSF9 can augment CD8 + T cell-mediated tumor-related immune responses by boosting the immune functions of CD4 + T and NK cells [44, 45]. Meanwhile, the immune status of tumors are related to the TNFRSF9 expression levels in gastric cancer. If tumor cells exhibit high expression of TNFRSF9, the body's ability to kill these tumor cells will be stronger [46]. Additionally, the combined use of TNFRSF9 agonists and PD-L1 inhibitors can activate tumor-specific cytotoxic T cells, thereby improving tumor killing [47]. In our MR analysis, we discovered that genetically predicted circulating levels of TNFRSF9 act as protective factors against thyroid cancer. Additionally, the downregulation of circulating TNF levels may participate in the downstream processes of thyroid cancer. Our MR study indicates that targeting TNFRSF9, combined with current immunotherapies, holds significant potential for the treatment of thyroid cancer.

There remains ongoing debate concerning the relationship between TNFSF14 and cancer. Some studies suggest that TNFSF14 can sensitize tumors to IFN-γ-mediated apoptosis, induce the normalization of tumor vasculature [48–50], stimulate effector cell functions, and promote the infiltration of CD8 + T cells into tumors, which helps establish long-term anti-tumor memory [51–54]. Conversely, some studies have demonstrated that the overexpression of TNFSF14 is associated with poorer clinicopathological characteristics and adverse prognosis [55–57]. Our study demonstrates that TNFSF14 is positively associated with the risk of thyroid cancer, and this association can be mediated by the levels of Lactosyl-N-palmitoyl-sphingosine (d18:1/16:0) and X-12013 in the blood, with mediation proportions of 8.55% and 5.78%, respectively. Current research suggests that Lactosyl-N-palmitoyl-sphingosine (d18:1/16:0) levels have significance in predicting inflammatory bowel disease and gestational diabetes [58, 59], but no studies are related to cancer. Additionally, the role and mechanisms of X-12013 in cancer are also unclear. Therefore, further molecular experiments need to investigate the roles and specific mechanisms of Lactosyl-N-palmitoyl-sphingosine (d18:1/16:0) and X-12013 in thyroid cancer. Therefore, future research should first conduct in vitro and in vivo experiments to verify the expression differences of these inflammatory cytokines and blood metabolites between thyroid cancer patients and healthy individuals. Subsequently, these expression differences could serve as potent tools for the early diagnosis and identification of thyroid cancer. Finally, TNFSF14 or these mediating blood metabolites could be targeted therapeutically by developing inhibitors specifically directed at these inflammatory cytokines or blood metabolites, thereby aiding in the treatment of thyroid cancer and improving the prognosis of patients.

CD244, an immunoregulatory transmembrane receptor, presents potential for immunotherapy [60]. Current studies indicate that CD244 primarily transmits inhibitory signals in tumor-associated immune cells, resulting in immune cell dysfunction. Immune checkpoint inhibitors are rendered ineffective or develop resistance as a result of this dysfunction [61–66]. Consistent with the aforementioned findings, our MR analysis also suggests that increased circulating CD244 levels are correlated with a higher thyroid cancer risk. Therefore, in future thyroid cancer treatments, strategies targeting CD244 inhibition may serve as an additional approach to existing immune checkpoint inhibitors or as a complement to conventional chemotherapy approaches.

Fibroblast Growth Factors (FGFs) stimulate or maintain cellular functions on metabolism, tissue homeostasis, and development through the signaling axis mediated by fibroblast growth factor receptors (FGFRs). FGF19, in particular, is involved in bile acid homeostasis, glucose and lipid metabolism, and energy expenditure [67]. An observational study has also indicated that circulating FGF19 levels are reduced in individuals with hypothyroidism and subclinical hypothyroidism [68]. However, there are limited experiments and studies related to FGF19 and cancer. Hu et al. highlighted that high FGF19 expression is linked to poor prognosis in advanced serous ovarian cancer [69]. In our MR analysis, genetically predicted circulating levels of FGF19 have been identified as a protective factor for thyroid cancer. Future experiments are necessary to explore the causality between FGF19 and cancer further, to determine its potentials in therapeutic target for cancer, particularly thyroid cancer.

C–C motif chemokines can participate in inflammatory responses. They are capable of regulating recruitments of various cells within the tumor microenvironment [70]. CCL19 is a crucial chemotactic factor involved in regulating both physiological conditions and pathological situations, including cancer [71, 72]. However, the upregulation or downregulation of CCL19 in cancer varies depending on the specific tumor microenvironment and remains uncertain [73]. Several studies have observed the immunostimulatory and antitumor functions of CCL19 [74–76], while other studies have highlighted its role in inhibiting tumor cell apoptosis and promoting metastasis [77–79]. Our MR analysis suggests that genetically predicted circulating CCL19 has a protective role against thyroid cancer. Consequently, the specific effects of CCL19 on thyroid cancer should be examined in the specific characteristics and interactions within the thyroid cancer tumor microenvironment.

IL2RB, referred to as CD122, has been demonstrated in studies to be activated by endogenous IL2 or through biased therapeutic stimuli. This activates CD8 + , CD4 + , and NK cells [80]. As a result, it has the potential to become a promising target for therapy and is studied in phase II/III clinical trials in conjunction with immune checkpoint blockade to treat advanced solid tumours [81]. In our MR analysis, genetically predicted circulating IL2RB has been identified as a protective factor against thyroid cancer, aligning with existing research findings. However, research on IL2RB and cancer treatment remains relatively limited. Future experiments are necessary to further investigate the association between IL2RB and cancer, in order to assess its potential in therapeutic target of thyroid cancer.

Human S100A12 is almost secreted by neutrophil granules. It participates in the innate immune response, related to some autoimmune reactions. The S100A12 is markedly overexpressed at sites of inflammation, and elevated serum S100A12 levels were detected in individuals with inflammatory, neurodegenerative, metabolic, and neoplastic diseases. It has been indicated that S100A12 holds significant potential as a sensitive diagnostic marker for local inflammatory processes [82]. Currently, research on the correlation between S100A12 and cancer are limited. Contrary to previous research findings, our MR analysis indicates that genetically predicted circulating S100A12 serves as a protective factor against thyroid cancer. Future research is needed to determine potentials of S100A12 as a tumor marker or therapeutic target.

FLT3LG, a growth factor, binds to Fms-like tyrosine kinase 3 on dendritic cells, promoting their differentiation and expansion. The beneficial effect of FLT3LG in stimulating dendritic cells, enhancing cancer sensitivity to immunotherapy has been demonstrated. Also, elevated FLT3LG levels are closely linked to improved progression-free survival, primarily due to reduced metastatic risks [83, 84]. Meanwhile, a clinical study on cervical cancer found that decreased FLT3LG expression is linked to a poor prognosis [85]. This aligns with the findings of our MR analysis, which determined that genetically predicted circulating FLT3LG acts as a protective factor against thyroid cancer. Consequently, it is essential to investigate the feasibility of combining FLT3LG with immunotherapy methods and to elucidate relationships between FLT3LG and the prognosis of thyroid cancer patients in future studies.

IFN-γ is crucial in activating cellular immunity and stimulating antitumor immune responses [86]. It functions by promoting cell quiescence, apoptosis, and antiproliferation. By inhibiting tumor angiogenesis, activating pro-inflammatory macrophages, and causing regulatory T cells to die, IFN- may be used to prevent tumor growth [87, 88]. Existing studies indicate that IFN-γ is an anti-cancer factor capable of inhibiting the proliferation of papillary thyroid carcinoma cells [89]. Our MR analysis indicates that the downregulation of circulating IFN-γ may be related to downstream processes of thyroid cancer, thus further experiments are necessary to validate this hypothesis. The LIFR promotes the pluripotency of stem cells and regulates their proliferations and differentiations [90]. LIFR is highly expressed in melanoma, nasopharyngeal carcinoma, and prostate cancer, and represents poor prognosis [91–93]. Our MR analysis suggests that the upregulation of LIFR levels may participate in the downstream development processes of thyroid cancer, and this conclusion needs to be further confirmed.

First, we employed various strategies to reveal the bidirectional causality between 91 inflammatory cytokines and thyroid cancer from a genetic perspective. The types of inflammatory cytokines involved are more diverse than those in previous MR studies, and the results of various strategies complemented each other. Additionally, we examined the heterogeneity and horizontal pleiotropy, utilized the MR-PRESSO method to identify outliers, and applied stringent Bonferroni correction, significantly enhancing the reliability of our results. Furthermore, the GWAS data originated from different consortia, reducing potential errors from sample overlap. Lastly, we introduced data on 1400 blood metabolites to explore the mediating role between blood metabolites, specific inflammatory cytokines, and the causal relationship with thyroid cancer.

However, there are limitations of our research, including potential MR errors and the inherent constraints of MR statistics. Therefore, further studies are necessary to validate the conclusions derived from MR statistical analyses. It is important to point out that the Bonferroni correction is a relatively stringent method for multiple testing correction. Although corrected positive results are more reliable, this method may elevate the false negative rate, potentially missing some true causal effects. For identifying SNPs associated with inflammatory cytokines and blood metabolites, we set a relatively lenient yet still significant threshold of 5 × 10^–6^, drawing on past experiences to obtain an adequate number of SNPs. Despite the removal of LD and exclusion of SNPs with an F-statistic below 10, we must recognize that relaxing the threshold for identifying SNPs inevitably carries the risk of introducing false associations. Additionally, the genetic variations in our study might explain only a small fraction of the overall risk factors. To reduce data heterogeneity and ensure result reliability, we selected GWAS data from the European population for analysis and conducted sensitivity analyses along with funnel plot visualizations. Genetic background, environmental factors, and their interactions across different populations may cause SNPs to exert varying effects on exposure and outcomes. These differences pose a significant challenge in managing GWAS data heterogeneity across different biobanks or populations and limit the generalizability of our findings to populations outside Europe. As previously mentioned, differences in genetic background and living environments among populations are considerable. Finally, it must be acknowledged that although the metabolomics in cancer diagnosis and treatment is increasingly widespread, current research on metabolites and cancer is not comprehensive, especially in the field of thyroid cancer. Therefore, the discussion section of this paper, after summarizing our MR analysis results, mainly uses inflammatory cytokines as an entry point to connect existing research with our MR analysis results, and looks forward to the clinical value of these MR analysis results for future thyroid cancer diagnosis and treatment. In the future, we hope that more research will be done on the relationship and mechanisms between inflammatory cytokines, blood metabolites, and thyroid cancer.

Our study determined the causalities between inflammatory cytokines, blood metabolites, and thyroid cancer through MR analysis, identifying a group of inflammatory cytokines and blood metabolites that can cause the thyroid cancer, uncovering the mediating effect of specific blood metabolites between TNFSF14 and thyroid cancer, highlighting the critical roles of inflammatory and metabolic pathways in the pathogenesis of thyroid cancer. These findings suggest that specific inflammatory cytokines and blood metabolites can serve as biomarkers for early identification and risk stratification of thyroid cancer, and may also become potential targets for cancer therapy, providing genetic evidence for future advancements in thyroid cancer detection and treatment.

## Supplementary Information

Additional file 1

Additional file 2

## Data Availability

This study analyzed publicly accessible datasets, for which we have properly attributed the original research in the body of the paper. The relevant GWAS data can be accessed at the following link: https://www.ebi.ac.uk/gwas/.
