# Supplementary material for: The causal relationships between inflammatory cytokines, blood metabolites, and thyroid cancer: a two-step Mendelian randomization analysis
Source: Discov Oncol. 2025 Mar 12;16:301. doi: 10.1007/s12672-025-02029-w (PMC11904021; doi:10.1007/s12672-025-02029-w)
Supplement: Supplementary file 1 — Additional file 1 [file 12672_2025_2029_MOESM1_ESM.docx]

**Supplementary Materials:**

**Supplementary Table:**

**Supplementary Table 1**: Selected circulating inflammatory cytokines-associated SNPs

**Supplementary Table 2**: Selected blood metabolites-associated SNPs

**Supplementary Table 3**: Selected thyroid cancer-associated SNPs

**Supplementary Table 4**: Selected TNFS14-associated SNPs

**Supplementary Table 5**: Causal effects of inflammatory cytokines on thyroid cancer

**Supplementary Table 6**: Causal effects of thyroid cancer on inflammatory cytokines

**Supplementary Table 7**: Causal effects of blood metabolites on thyroid cancer

**Supplementary Table 8**: Causal effects of TNFSF14 on specific blood metabolites

**Supplementary Table 9**: Associations of inflammatory cytokines with thyroid cancer in heterogeneity tests

**Supplementary Table 10**: Associations of inflammatory cytokines with thyroid cancer in pleiotropy tests

**Supplementary Table 11**: Associations of thyroid cancer with inflammatory cytokines in heterogeneity tests

**Supplementary Table 12**: Associations of thyroid cancer with inflammatory cytokines in pleiotropy tests

**Supplementary Table 13**: Associations of blood metabolites with thyroid cancer in heterogeneity tests

**Supplementary Table 14**: Associations of blood metabolites with thyroid cancer in pleiotropy tests

**Supplementary Table 15**: Associations of TNFSF14 with specific metabolites in heterogeneity tests

**Supplementary Table 16**: Associations of TNFSF14 with specific metabolites in pleiotropy tests
